# Clinical profile of parkinsonism and Parkinson's disease in Lagos, Southwestern Nigeria

**DOI:** 10.1186/1471-2377-10-1

**Published:** 2010-01-05

**Authors:** Njideka U Okubadejo, Oluwadamilola O Ojo, Olajumoke O Oshinaike

**Affiliations:** 1 Department of Medicine, College of Medicine, University of Lagos, Nigeria; 2 Neurology Unit, Lagos University Teaching Hospital, Idi Araba, Lagos State, Nigeria; 3 Lagos State University College of Medicine, Ikeja, Lagos State, Nigeria

## Abstract

**Background:**

Current data on the pattern of parkinsonism and Parkinson's disease in Nigerians are sparse.

This database was designed to document the clinical profile of PD in Nigerians, and compare this to prior observations.

**Methods:**

A database of patients presenting to the Neurology out-patients clinic of the Lagos University Teaching Hospital was established in October 1996. Demographic and clinical data at presentation (disease stage using Hoehn and Yahr scale; 'off' state severity on the Unified Parkinson's disease Rating Scale) were documented for patients diagnosed with parkinsonism between October 1996 and December 2006. Cases were classified as Parkinson's disease or secondary parkinsonism (in the presence of criteria suggestive of a secondary aetiology).

**Results:**

The hospital frequency of parkinsonism (over a 2-year period, and relative to other neurologic disorders) was 1.47% (i.e. 20/1360). Of the 124 patients with parkinsonism, 98 (79.0%) had PD, while 26 (21.0%) had secondary parkinsonism. Mean age (SD) at onset of PD (61.5 (10.0) years) was slightly higher than for secondary parkinsonism (57.5 (14.0) years) (P = 0.10). There was a male preponderance in PD (3.3 to 1) and secondary parkinsonism (2.7 to 1), while a positive family history of parkinsonism was present in only 1.02% (1/98) of PD. There was a modestly significant difference in age at onset (SD) of PD in men (60.3 (10.4)) compared to women (65.2 (7.9)) (T = 2.08; P = 0.04). The frequency of young onset PD (≤ 50 years) was 16.3% (16/98). The mean time interval from onset of motor symptoms to diagnosis of PD was 24.6 ± 26.1 months with majority presenting at a median 12 months from onset. On the H&Y scale, severity of PD at presentation was a median 2.0 (range 1 to 4). PD disease subtype was tremor-dominant in 31 (31.6%), mixed 54 (55.1%) and akinetic-rigid 14 (14.3%). Hypertension was present as a co-morbidity in 20 (20.4%), and diabetes in 6 (6.12%).

**Conclusions:**

The clinical profile of PD in Nigerians is similar to that in other populations, but is characterized by delayed presentation as has been reported in other developing countries. Young-onset disease occurs but may be less commonly encountered, and frequency of a positive family history is lower than in western populations.

## Background

Parkinson's disease (PD) is a slowly progressive neurodegenerative movement disorder resulting from selective loss of nigral dopaminergic neurons of unknown aetiology. PD predominantly affects the elderly population, and is clinically characterized by both motor and non-motor manifestations. The disease occurs worldwide, with studies in Africa yielding hospital frequencies of 0.3 to 2.3% of neurological diseases [1]. In western Nigeria, the prevalence rate is between 50 - 90 per 100,000 of the population aged above ten years [2,3]. The lower prevalence in comparison to both the black and Caucasian population in industrialized western countries such as the United States of America (USA) has been attributed to possible genetic, environmental or demographic factors [4].

The pattern of occurrence of parkinsonism (including PD) in Nigerians was comprehensively reviewed by earlier authors in Ibadan in 1979 [5], providing a basis for comparison with observed patterns globally. Their findings compared favourably with the earlier reports of Hoehn and Yahr published 12 years prior and based on cases seen in the USA [6]. Subsequently, there has been a void in reports of the pattern of PD observed in sub-Saharan African populations in recent years, particularly as the continent becomes more industrialized. The catchment area of the Lagos University Teaching Hospital, a tertiary health institution and referral center for neurological conditions, includes (but is not limited) to Lagos State in Southwestern Nigeria (a predominantly urban and industrialized area) and its environs.

This study provides a description of the clinical characteristics of our cohort of Nigerian PD patients, and provides a basis for comparison to existing observations, so as to determine if there has been any appreciable variation from previously reported patterns.

## Methods

A database of patients presenting to the Neurology out-patients clinic of the Lagos University Teaching Hospital was established in October 1996. Approval of the study protocol was obtained from the Health Research and Ethics Committee of the LUTH, and informed consent was obtained from all patients. Demographic and clinical data of all consecutively attending patients with parkinsonism over a ten year period (October 1996 to December 2006) were systematically documented. The definition of the syndrome of parkinsonism was based on a combination of clinical data and presumed aetiology. The diagnosis of parkinsonism was made on the basis of the presence of at least three of the four cardinal features *i.e. tremors, rigidity, bradykinesia, and postural or gait abnormality*. All patients diagnosed as PD had all 3 of tremors, rigidity, and bradykinesia. The diagnosis of idiopathic parkinsonism (Parkinson's disease) was premised on the presence of parkinsonism without any identifiable secondary cause, asymmetry at onset, absence of atypical features (e.g. early prominent dysautonomia, early or preceding cognitive impairment, cerebellar features, muscle weakness, temporal relationship to drug or toxin exposure, bilateral symptoms at onset, lower-body parkinsonism, otherwise unexplained corticospinal tract signs), and, in retrospect, sustained levodopa responsiveness.

The diagnosis of the specific secondary parkinsonism was based on the constellation of clinical features suggestive of a secondary aetiology [6-8]. Neuroimaging (brain computerised tomographic scan and magnetic resonance imaging) was available for 9 cases including the patients with olivopontocerebellar atrophy (1), hemiparkinsonism (1), 3 with vascular parkinsonism secondary to cerebrovascular disease, and 4 with PD.

A standard proforma was used to document the diagnosis of PD, personal data (present age and sex) as well as the patient characteristics (age at onset of the disease, age at presentation, duration of illness prior to presentation, and initial symptoms and distribution of symptoms of PD). The age at onset of PD-related motor symptoms was used to define age at onset of the disease in this study. Young onset PD was defined as age at onset ≤ 50 years. A positive family history of PD was defined as a history of parkinsonian features or reported physician's diagnosis of PD in a first degree relative. The severity at presentation was classified using the Hoehn and Yahr (H&Y) scale [9,10]. PD cases presenting after December 2004 were routinely assessed in the *'off' state *at presentation with the Unified Parkinson's Disease Rating Scale (UPDRS) version 3.0 (11).

Data were analyzed using EPI Info^® ^2002 statistical software. Continuous data are expressed as mean (standard deviation) (SD), median, and ranges. Mean values were compared using the Student t test. The X^2 ^test was used to compare categorical variables which are presented as proportions (frequency %). P < 0.05 was considered as statistically significant.

## Results

### Hospital frequency of parkinsonism and PD

The hospital frequency of parkinsonism with respect to other neurologic disorders seen in the outpatient neurology clinic of the LUTH was determined by comparing total number of new referrals with parkinsonism seen over a two year period (January 2005 to December 2006) to total number of new outpatients attending the adult neurology clinic in the same period. The adult neurology clinic attends to patients aged 12 years and above. In the two-year period, the total number of new cases of parkinsonism was 24 and total number of new referrals was 1360, giving a hospital frequency of 1.76%. The corresponding hospital frequency of PD was 1.47% (i.e. 20/1360).

### Classification of parkinsonism (relative frequencies of PD and secondary parkinsonism)

A total of 124 patients fulfilling the diagnostic criteria for the syndrome of Parkinsonism were seen during the ten-year period from 1996 to 2006. Of these, 98 (79.0%) had features compatible with PD, while 26 (21.0%) had secondary parkinsonism as shown in Table 1.

**Table 1:** Comparison of the demographic and clinical characteristics of cases with PD and secondary parkinsonism

Characteristic	Parkinson's disease n = 98	Secondary parkinsonism n = 26	Statistical comparison
Frequency (%)	98/124 (79.0%)	26/124 (21.0%)	
Male (%)	75 (76.5%)	19 (73.1%)	RR = 1.04 (95% CI 0.83-1.30)
Female (%)	23 (23.5%)	7 (26.9%)	
Male to female ratio	3.3 to 1	2.7 to 1	
Mean age (SD) at onset, years	61.5 (10.0)	57.5 (14.0)	T = 1.66; P = 0.10
Median age at onset	63	61.5	
Age range at onset	37 to 77	22 to 78	
Family history of PD in FDR	1 (1.02%)	0 (0%)	RR = 1.27 (95% CI 1.16-1.39)

FDR First degree relative

RR Relative risk

CI 95% Confidence Interval

### Comparison of clinical characteristics of PD and secondary parkinsonism

The age at onset of symptoms in the 98 PD cases (mean(SD) 61.5(10.0) years, median 63, range 37 to 77) was slightly but insignificantly higher than that of the 26 patients with secondary parkinsonism overall (mean(SD) 57.5(14.0)) years, median 61.5, range 22 to 78) (T = 1.66; P = 0.10) (Table 1). However, the 5 patients with drug-induced parkinsonism were significantly younger than those with PD (mean (SD) 46.0(11.3) years, median 45.0, range 37 to 65) (T = 3.34; P = 0.001).

There was a male preponderance in both idiopathic PD (male to female ratio 3.3 to 1) and secondary parkinsonism (2.7 to 1 overall, 4.25 to 1 for secondary parkinsonism excluding drug-induced, and 1 to 1.5 for drug-induced parkinsonism). A positive family history of parkinsonism was present in only 1.02% (1/98) of PD cases and 0% of secondary parkinsonism. The frequencies of the presumed secondary aetiologies of parkinsonism are shown in Table 2.

**Table 2:** Aetiologies of secondary parkinsonism in Lagos, Nigeria

Presumed aetiology	Number	Frequency %
Vascular parkinsonism	9	34.6
Drug-induced parkinsonism	5	19.2
Multiple System Atrophy	4	15.4
Lewy body dementia	3	11.5
Toxin exposure (carbon-monoxide poisoning)	1	3.8
Progressive supranuclear palsy	1	3.8
Hemiparkinsonism - hemiatrophy	1	3.8
Juvenile parkinsonism with dystonia and hemiatrophy	1	3.8
Primary amyloidosis with parkinsonism	1	3.8

* MSA - Shy Drager (autonomic) = 3; MSA - Olivopontocerebellar atrophy = 1

### Clinical characteristics of PD

#### Age at onset and frequency of young onset PD

There was a modestly significant difference in age at onset of PD in men (mean(SD) 60.3 (10.4); median 62.0; range 37 to 77) compared to women (mean(SD) 65.2(7.9); median 68.0; range 41 to 74) (T = 2.08; P = 0.04). The frequency of young onset PD (≤ 50 years) was 16.3% (16/98), and was higher in men (15/75 i.e. 20%) compared to women (1/23 i.e. 4.3%) (Fisher exact test; P = 0.06). A positive family history of PD was present in 1/16 (6.2%) of the young onset PD, while none of the PD patients with age at onset above 50 years had a family history of PD.

#### Disease duration and severity at presentation

The mean (SD) time interval from the onset of motor symptoms to diagnosis of PD was 24.6 (26.1) months (men 24.6(28.4); female 24.4(17.2); T = 0.05, P = 0.96) with most patients presenting at a median 12 months from onset (range 4 to 156 months).

None of the patients had received a definite diagnosis of PD prior to specialist consultation. The diagnosis stated in the referral letter was as follows: neurological disorder (or neurological symptoms, not otherwise specified) - 40 (40.8%), tremors (cause unknown) - 33 (33.7%), parkinsonism - 10 (10.2%), stroke - 10 (10.2%), and osteoarthritis - 5 (5.1%).

Median H&Y scale score of PD patients at presentation was a median 2.0 (range 1 to 4; mean(SD) 2.3(0.8)). The distribution of PD cases by disease severity at presentation was as follows: stage 1 - 12 (12.2%), stage 2 - 53 (54.1%), stage 3 - 29 (29.6%), stage 4 - 4 (4.1%), stage 5 - 0 (0%).

#### Clinical features of PD cases evaluated between 1996 and 2006

The initial side of onset of the earliest PD-related motor symptoms in our cases was the right side (60/98 i.e. 61.2%), whereas the left side was the side of onset in 38 cases (38.8%). The upper limb was initially affected in 87 (88.8%) while the lower limb was the initial site in 11 (11.2%). The first motor symptom was reported as tremors in 75 (76.5%), and as stiffness/persistent aching/rigidity in 23 (23.5%). Disease subtype was characterised as tremor-dominant in 31 (31.6%), mixed in 54 (55.1%) and akinetic-rigid in 14 (14.3%). Hypertension was present as a co-morbidity in 20 (20.4%), and diabetes in 6 (6.12%). UPDRS scores were included as part of routine evaluation of all PD cases in the later years of the study and are available for 40 consecutively presenting cases presenting after December 2004. For the 40 cases (median HY scale 2; mean (SD) 2.3(0.9)), the UPDRS scores (total, motor and ADL) are shown in Table 3, along with the summary data of other clinical characteristics of all 98 PD cases.

**Table 3:** Clinical profile of PD at presentation in Nigerians (1996 to 2006)

Clinical characteristic	Mean (SD)	Median	Range
Age at onset of PD in men, years	60.3 (10.4)	62.0	37 - 77
Age at onset of PD in women, years	65.2 (7.9)	68.0	41 - 74
Interval from onset to diagnosis, months	24.6 (26.1)	12.0	4 - 156
Hoehn & Yahr scale	2.3 (0.8)	2.0	1 - 4
UPDRS Total score (n = 40)	57.7 (25.0)	55.5	19 - 128
UPDRS Motor score (n = 40)	41.1 (17.6)	42.0	9 - 87
UPDRS ADL score (n = 40)	13.6 (7.2)	11.0	4 - 36

Total number of PD cases = 98 unless otherwise stated

UPDRS Unified Parkinson's Disease Rating Scale version 3.0

ADL Activities of daily living

#### Medication use in PD cases

All the 98 PD cases (100%) were placed on levodopa/carbidopa 250 mg/25 mg starting at 1/2 tablet (125 mg levodopa/12.5 mg carbidopa) 8 hourly with individualized upward titration and dose adjustments based on clinical response in each case. Of these 98, medication utilization data are available for 75 PD cases still attending the out-patient clinic as at December 2006 (dropouts due to either death, relocation or loss to follow-up). All 75 cases (100%) were on levodopa/carbidopa (LD/CD). The distribution was as follows: LD/CD monotherapy - 34 (45.3%), LD/CD + dopamine agonist - 21 (28%); LD/CD + anticholinergic benztropine - 12 (16%), and LD/CD + dopamine agonist + anticholinergic benztropine - 8 (10.7%). The mean LD dose (mg/24 hours) (SD) was 605.8(181.8) (range 375 to 1125). The dopamine agonist used in 28/29 cases was bromocriptine, while only 1 PD patient was on ropinrole.

## Discussion

This study provides a description of the current clinical profile of PD and secondary parkinsonism as seen over the last ten years at a tertiary referral centre in South-western Nigeria. The spectrum of cases encountered may thus be subject to referral bias, with a tendency to see more severe or more complex neurological cases. The study however provides data for comparison with an earlier report and global perspectives of parkinsonism and PD.

In contrast to the only published report on the clinical characteristics of parkinsonism in Nigerians from Osuntokun and Bademosi [5], PD accounted for a significantly higher proportion of causes of parkinsonism in the present study (79% compared to 38%). The current relative frequency of PD in our study is in keeping with available literature, which ranks PD as causing approximately three quarters of all cases of parkinsonism. The apparent disparity may be accounted for by more recent improvements and streamlining of the diagnostic criteria for PD and other parkinsonian syndromes in both the clinical and research arena in contrast to the status at the time of the earlier report [5,12-14].

The clinical profile of our patients differed slightly from that reported in the earlier Nigerian study. First, the mean age at onset in the present study was 61.5 years, higher than the 55.6 years reported by Osuntokun *et al *[5]. Both studies represent patients seen in the same geographical region, with both tertiary centres being within two hours' drive of each other. The increase in age at onset may thus be an accurate phenomenon akin to that observed by Hoehn and Yahr in which they found a time trend of increasing age at onset over decades of study [9]. It has been documented that as populations age, the age at onset of PD tends to increase. The male preponderance reported in the earlier study (ratio 4.5 to 1) was also documented here, but the magnitude in this study was lower (ratio 3.3 to 1). Although this male predilection appears to be a consistent finding, the precise reason is unknown, and the possibility of a neuroprotective effect mediated by estrogen in women exists [15-17]. Experimental evidence indicates that estrogen may mediate this effect via several mechanisms including inhibition of dopamine transporter affinity and prevention of entry of neurotoxic agents into dopaminergic nerve terminals, thereby reducing nigrostriatal degeneration [17].

Genetic contributions to the aetiology of PD are undisputable, with transmission as an autosomal recessive or dominant trait linked to mutations in several genes, including α-synuclein, parkin, DJ1, leucine-rich repeat kinase (LRRK) 2, etc [18,19]. Genetic susceptibility appears to be more apparent (although not exclusive) in young onset PD compared to persons developing the disease after the 6^th ^decade of life. We found a positive family history of PD in a first degree relative in only one of our patients who incidentally had young onset PD starting below age 50. Young onset PD was documented in 16.3% of our cohort, with a five-fold higher frequency in men (20%) compared to women (4.3%). We acknowledge the fact that the number of PD cases reported here (including only 23 females) is small and may account for the low rates of a positive family history and gender disparity in frequency of young-onset PD; our findings are thus subject to further validation. A preliminary analysis of the genetic contributions to PD in Nigerians (which included some of the cases reported in this present study), explored the role of mutations in *LRRK2*, *PRKN *and *ATXN3 *in apparently sporadic PD compared to age - and ethnically-matched controls. The study found that common pathogenic mutations in these genes, previously observed in several populations, are not a frequent cause of PD in Nigerians [20].

Overall, the clinical profile of PD in Nigerians does not appear to vary substantially from disease characteristics reported in other populations. Delayed presentation (and late referral) are not germaine to this study population and is one of the challenges encountered in managing PD in Africa [21]. Poor recognition of the cardinal features of parkinsonism and of the existence or benefit of available therapies in alleviating the symptoms and improving the quality of life of people with PD may contribute to late referrals. This has further implications as it will delay utilization of disease-modifying strategies which may become available in the future. Strategies to improve early recognition and referral include strengthening undergraduate movement disorders curriculum, and improving public awareness as to the existence, cardinal features, and treatment options of parkinsonism via the media. The need for such an approach is strengthened by the increased likelihood of physicians encountering PD and other neurodegenerative diseases of the elderly in the future in developing countries experiencing an epidemiologic transition marked by aging of the population.

We acknowledge the methodological limitations of this study in that follow-up data on the disease course and rate of progression, and an objective measure of magnitude of response to levodopa therapy, and mortality data which would enhance insight into the phenotypy of our PD cohort are lacking. An earlier published study of a subgroup of this cohort (28 PD cases and 28 age-matched controls) initially seen between January and June 1997 and followed up between January and May 2003 (after a 6-year interval) reported a case fatality rate of 25% in PD, compared to 7.1% in controls [22]. The peculiarity of our clinical practice scenario, characterized by the availability of a very narrow spectrum of antiparkinsonian medications and dosage formulations is reflected in the distribution of medications used in PD treatment in our cohort. We also emphasize that this factor would also limit the conclusiveness of any description of treatment outcomes in our cohort at the present time.

## Conclusion

Our study provides data on the clinical profile of Nigerian patients with PD and demonstrates that it is similar to that from other populations, but is characterized by delayed presentation as has been reported in other developing countries. Young-onset disease occurs but may be less commonly encountered, and frequency of a positive family history is lower than in western populations.

## Abbreviations

ADL: Activities of Daily Living; H&Y: Hoehn and Yahr; PD: Parkinson's disease; UPDRS: Unified Parkinson's Disease Rating Scale; WHO: World Health Organization; SD: Standard deviation.

## Competing interests

The authors declare that they have no competing interests.

## Authors' contributions

NUO conceptualized the study. NUO, OOOj and OOOs obtained the data. NUO conducted the data analysis. NUO, OOOj and OOOs participated in drafting the initial manuscript, review of the manuscript for intellectual content, and approval of the final manuscript.

## Pre-publication history

The pre-publication history for this paper can be accessed here:

http://www.biomedcentral.com/1471-2377/10/1/prepub

## References

[B1] Okubadejo NU Bower JH Rocca WA Maraganore DM Parkinson's disease in Africa: a systematic review of epidemiologic and genetic studies Mov Disord 2006 12 2150 2156 10.1002/mds.21153 17044056

[B2] Osuntokun BO Schoenberg BS Nottidge VA Adeuja A Kale O Adeyefa A Bademosi O Olumide A Oyediran ABO Pearson CA Bolis CL Research protocol for measuring the prevalence of neurologic disorders in developing countries: results of a pilot study in Nigeria Neuroepidemiology 1982 1 143 153 10.1159/000110696

[B3] Osuntokun BO Adeuja AO Schoenberg BS Bademosi O Nottidge VA Olumide AO Ige O Yaria F Bolis CL Neurological disorders in Nigerian Africans: a community-based study Acta Neurol Scand 1987 75 13 21 10.1111/j.1600-0404.1987.tb07883.x 3033973

[B4] Schoenberg BS Osuntokun BO Adeuja AO Bademosi O Nottidge V Anderson DW Haerer AF Comparison of the prevalence of Parkinson's disease in black populations in the rural United States and in rural Nigeria: door-to-door community studies Neurology 1988 38 645 646 3352927 10.1212/wnl.38.4.645

[B5] Osuntokun BO Bademosi O Parkinsonism in the Nigerian African: a prospective study of 217 patients East Afr Med J 1979 56 597 607 520239

[B6] Quinn N Multiple system atrophy - the nature of the beast J Neurol Neurosurg Psychiatry 1989 52 78 89 10.1136/jnnp.52.Suppl.78 PMC1033311 2666581

[B7] Ahlskog JE Diagnosis and differential diagnosis of Parkinson's disease and parkinsonism Parkinsonism and Related Disorders 2001 7 63 70 10.1016/S1353-8020(00)00047-X 11008198

[B8] Schrag A Ben-Shlomo Y Quinn N How valid is the clinical diagnosis of Parkinson's disease in the community? J Neurol Neurosurg Psychiatry 2002 73 529 534 10.1136/jnnp.73.5.529 12397145 PMC1738115

[B9] Hoehn MM Yahr MD Parkinsonism: onset, progression and mortality Neurology 1967 17 427 442 6067254 10.1212/wnl.17.5.427

[B10] Goetz CG Poewe W Rascol O Sampaio C Stebbins GT Counsell C Giladi N Holloway RG Moore CG Wenning GK Yahr MD Seidl L Movement Disorder Society Task Force on Rating Scales for Parkinson's Disease Movement Disorder Task Force report on the Hoehn and Yahr staging scale: status and recommendations Mov Disord 2004 19 1020 1028 10.1002/mds.20213 15372591

[B11] Movement Disorder Society Task Force on Rating Scales for Parkinson's Disease The Unified Parkinson's Disease Rating Scale (UPDRS): status and recommendations Mov Disord 2003 18 738 50 10.1002/mds.10473 12815652

[B12] Gibb WR Lees AJ The relevance of the Lewy body to the pathogenesis of idiopathic Parkinson's disease J Neurol Neurosurg Psychiatry 1988 51 745 52 10.1136/jnnp.51.6.745 2841426 PMC1033142

[B13] Suchowersky O Reich S Perlmutter J Zesiewicz T Gronseth G Weiner WJ Quality Standards Subcommittee of the American Academy of Neurology Practice Parameter: diagnosis and prognosis of new onset Parkinson disease (an evidence-based review): report of the Quality Standards Subcommittee of the American Academy of Neurology Neurology 2006 66 968 75 10.1212/01.wnl.0000215437.80053.d0 16606907

[B14] Alvarez MV Evidente VG Driver-Dunckley ED Differentiating Parkinson's disease from other parkinsonian disorders Semin Neurol 2007 27 356 62 10.1055/s-2007-985336 17701873

[B15] Bower JH Maraganore DM McDonnell SK Rocca WA Incidence and distribution of parkinsonism in Olmsted County, Minnesota, 1976-1990 Neurology 1999 52 1214 20 10214746 10.1212/wnl.52.6.1214

[B16] Baldereschi M Di Carlo A Rocca WA Vanni P Maggi S Perissinotto E Grigoletto F Amaducci L Inzitari D Parkinson's disease and parkinsonism in a longitudinal study: two-fold higher incidence in men. ILSA Working Group. Italian Longitudinal Study on Aging Neurology 2000 55 1358 1363 11087781 10.1212/wnl.55.9.1358

[B17] Dluzen DF Neuroprotective effects of estrogen upon the nigrostriatal dopaminergic system J Neurocytol 2000 29 387 99 10.1023/A:1007117424491 11424955

[B18] Pankratz N Foroud T Genetics of Parkinson disease Genet Med 2007 9 801 11 10.1097/GIM.0b013e31815bf97c 18091429

[B19] Biskup S Gerlach M Kupsch A Reichmann H Riederer P Vieregge P Wüllner U Gasser T Genes associated with Parkinson syndrome J Neurol 2008 255 Suppl 5 8 17 10.1007/s00415-008-5005-2 18787878

[B20] Okubadejo N Britton A Crews C Akinyemi R Hardy J Singleton A Bras J Analysis of Nigerians with apparently sporadic Parkinson disease for mutations in LRRK2, PRKN and ATXN3 PLoS ONE 2008 3 e3421 10.1371/journal.pone.0003421 18927607 PMC2559870

[B21] Dotchin CL Msuya O Walker RW The challenge of Parkinson's disease management in Africa Age Ageing 2007 36 122 7 10.1093/ageing/afl172 17261529

[B22] Okubadejo NU Ojini FI Danesi MA Longitudinal study of mortality predictors in Parkinson's disease in Nigerians African Journal of Medicine and Medical Sciences 2005 34 365 9 16752667

